# Causal relationship between gut microbiota and male erectile dysfunction: a Mendelian randomization analysis

**DOI:** 10.3389/fmicb.2024.1367740

**Published:** 2024-08-29

**Authors:** Shuaiqi Chen, Xiaolong Liu, Shangrong Wu, Guangyu Sun, Ranlu Liu

**Affiliations:** ^1^Department of Urology, The First Affiliated Hospital of Xinxiang Medical University, Xinxiang, China; ^2^Department of Urology, Tianjin Medical University General Hospital, Tianjin, China

**Keywords:** gut microbiota, erectile dysfunction, Mendelian randomization, GWAS, causal relationship

## Abstract

**Background:**

Several observational studies have reported an association between gut microbiota and male erectile dysfunction (ED). However, it remains unclear whether there is a causal relationship between gut microbiota and male ED. Thus, we aimed to investigate the potential causal relationship between gut microbiota and male ED through Mendelian randomization (MR) analysis.

**Objective:**

To assess the causal relationship between gut microbiota and male ED, we performed a two-sample MR analysis.

**Methods:**

We obtained gut microbiota genome-wide association studies (GWAS) data from the MiBioGen consortium and publicly available GWAS data on male ED from the OPEN GWAS database. Subsequently, we performed a two-sample MR analysis to evaluate the causal relationship between gut microbiota and male ED. Finally, we performed sensitivity analysis, including Cochran’s *Q* test, MR-Egger intercept analysis, MR-PRESSO, and leave-one-out analysis, to assess the level of heterogeneity and horizontal pleiotropy in the results.

**Results:**

Our MR analysis revealed a negative causal relationship between the genus Ruminococcaceae UCG013 and male ED (OR = 0.761, 95% CI 0.626–0.926), while the family Lachnospiraceae, genus Lachnospiraceae NC2004 group, genus Oscillibacter, and genus Tyzzerella3 may be associated with an increased risk of male ED, with the highest risk observed for family Lachnospiraceae (OR = 1.264, 95% CI 1.063–1.504). Furthermore, sensitivity analysis confirmed the reliability of our positive findings.

**Conclusion:**

Our MR analysis revealed a causal relationship between gut microbiota and male ED. This may contribute to a better understanding of the potential applications of gut microbiota in the occurrence and treatment of male ED.

## 1 Introduction

Erectile dysfunction (ED) is a common male condition characterized by the inability of the penis to achieve or maintain a sufficient erection for satisfactory sexual activity, lasting for at least 3 months (Salonia et al., 2021). ED significantly affects the physical and mental well-being of patients, creating immense pressure on family harmony and stability (Wang et al., 2023). Research indicates that ED is associated with various complications and related risk factors such as aging, smoking, obesity, cardiovascular diseases, and depression (Zeleke et al., 2021; Castela and Costa, 2016; Liu et al., 2018). Currently, phosphodiesterase type 5 inhibitors are the first-line treatment for ED, but the results are not satisfactory (Kang et al., 2023). With the development of technology, emerging targeted technologies such as stem cell therapy, protein therapy and low-intensity extracorporeal shockwave therapy (Li-ESWT), as well as intestinal probiotic therapies, are being utilized for the treatment of ED. However, emerging treatments are still in their infancy, and their pharmacological pathways and specific mechanisms have not yet been fully discovered (Kang et al., 2023; Chung et al., 2023).

Gut microbiota is a collective of a large number of bacteria that exist in the human gut. There are over 1,000 species of microbiota in the intestines of adults, with a total count exceeding 3.8 × 10^13^, roughly equivalent to the number of human cells (Sender et al., 2016). The gut microbiota exerts various effects on the host, including nutrient metabolism, immune regulation, and tumorigenesis (Durack and Lynch, 2019). Studies have also indicated that the gut microbiota can affect male fertility through mediating inflammation, substance metabolism, and psychological factors (Gorman and Golovanov, 2022; Zhang et al., 2022; Bear et al., 2021). In a cross-sectional study in Japan, a total of 192 male participants were enrolled and divided into two groups based on the International Index of Erectile Function (IIEF-5), and fecal 16S rRNA gene sequencing was performed on both groups. The result showed that the relative abundance of Alistipes and Clostoridium XVIII was significantly different between the two groups. Multivariate logistic analysis demonstrated that the relative abundance of Clostridium XVIII (OR, 2.06; 95% CI, 1.20–3.55, *p* = 0.009) and Alistipes (OR, 0.81; 95% CI, 0.66–0.99, *p* = 0.040), as well as the IPSS ≥8, were independent factors for low IIEF-5 (Okamoto et al., 2020). In another study, male mice that routinely consumed purified Lactobacillus initially isolated from human milk had enlarged testes and elevated serum testosterone levels. Mice with Lactobacillus neutrophils added to their drinking water showed a significant increase in the cross-sectional profile of the seminiferous tubules, spermatogenesis, and the number of Leydig cells per testis (Poutahidis et al., 2014). The current observational study found a strong relationship between ED and gut microbes, however, due to confounding factors and reverse causation, previous studies have not determined the exact causal relationship.

Mendelian randomization (MR) analysis MR utilized genetic variants significantly associated with exposure as instrumental variables (IVs) to infer causal relationships between exposure and outcome. The genotype corresponding to an individual’s IV locus reflects the level of exposure, while the parental allele is randomly assigned to the offspring during the deceleration period; thus, the MR approach, also known as a “natural randomized controlled trial,” avoids confounding bias and reverse causation, which are common in observational studies (Bowden and Holmes, 2019). Currently, The MR study by professor Fu found Desulfovibrionales and nine other bacteria to be associated with CKD, thus confirming that the gut microbiota plays an important role in the pathogenesis of CKD (Luo et al., 2023), while the study by Luo professor found a causal relationship between nine gut microbial taxa and cholelithiasis (Li et al., 2023). In our study, we initially explored the causal relationship between gut microbiota and ED through MR analysis.

## 2 Materials and methods

### 2.1 Study design and data source

**Figure 1:**
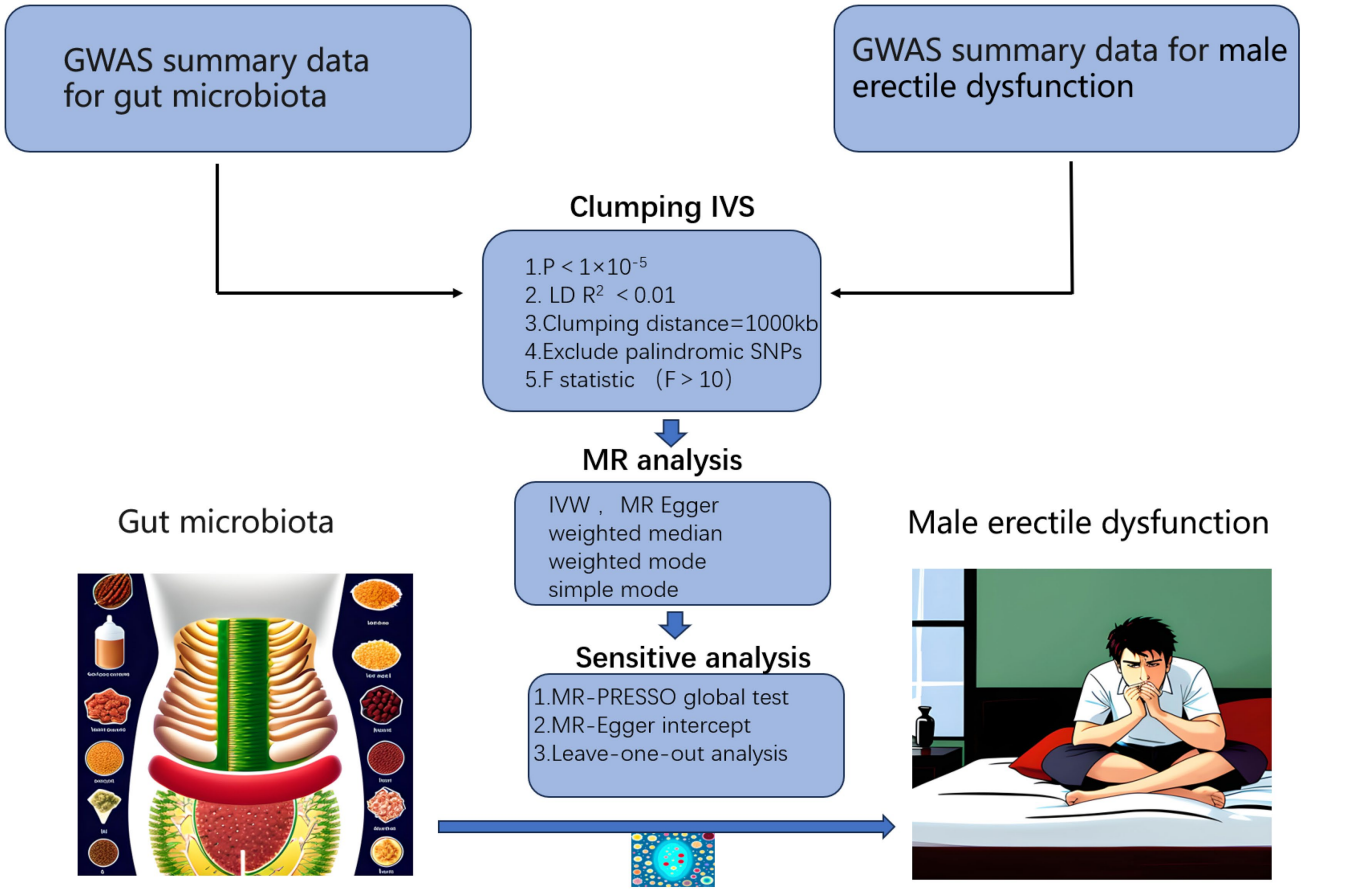
Workflow of our MR analysis. GWAS, genome-wide association studies; IVs, instrumental variables.

We performed a two-sample MR analysis in strict accord with the requirements of the STROBE-MR guidelines (Skrivankova et al., 2021), checklist was provided in Supplementary Table S1. The workflow of the study was illustrated in Figure 1, where gut microbiota was considered as the exposure factor and ED as the outcome. The statistical data of gut microbiota were obtained from 16S rRNA gene sequencing profiles and genotype data provided by MiBioGen, including a total of 18,340 participants from 24 cohorts, most of them were of European descent (13,266 individuals). This dataset includes 211 taxa, including 131 genera, 35 families, 20 orders, 16 classes, and 9 phyla (Kurilshikov et al., 2021). The GWAS data for ED were obtained from the study conducted by Bovijn et al. (2019), which included 6,175 cases and 217,630 controls from the Partners HealthCare Biobank, the Estonian Genome Center of the University, and the UK Biobank, all were of European descent. ED was defined as self-reported or physician-reported ED (ICD10 codes N48.4 and F52.2), or use of ED medications (sildenafil/Viagra, tadalafil/Cialis, or vardenafil/Levitra), or history of ED surgery (OPCS-4 codes L97.1 and N32.6).

### 2.2 Selection of instrumental variables

In our study, gut microbiota is considered as the exposure factor, and ED as the outcome. In MR studies, the selection of IVS follows three assumptions: IVS was strongly associated with exposure factors, not associated with outcomes and confounders. Additionally, the GWAS data were all approved by the Ethics Committee in the original form, so no additional access was required.

The following steps were taken to select SNPs: (1) due to the limited number of SNPs with genome-wide significance, a relatively loose significance threshold of *p* < 1 × 10^−5^ was used. (2) SNPs that violated the linkage disequilibrium (LD) criterion (*R*^2^ < 0.001, clumping distance = 10,000 kb) were excluded. (3) The *F*-statistic was calculated to assess the strength of SNPs. If the corresponding *F*-statistic was >10, it was considered that there was no significant weak instrumental bias. The formula for calculating *F* as follows: *F* = *R*^2^ × (*n* − 1 − *k*)/(1 − *R*^2^) × *k*. *R*^2^ represented the proportion of variance in the exposure explained by the genetic variants, *N* represented sample size, and *k* represented the number of instruments (Staiger and Stock, 1997). (4) SNPs with inconsistent alleles between exposure and outcome (e.g., A/G and A/C) and palindromic A/T or G/C alleles were excluded.

### 2.3 Statistical analysis

We performed five MR analysis methods, including the inverse variance weighting (IVW) method, the MR-Egger method, the weighted median method, the weighted mode method, and the simple mode method to assess the causal relationship between the gut microbiota and ED. IVW combines the Wald ratio estimates for each instrumental variable in a weighted linear regression of the instrumental variable on the outcome (Burgess et al., 2013). MR-Egger method was based on the instrument strength independent of direct effect (InSIDE) assumption, accounting for the presence of pleiotropy (Burgess et al., 2016). The weighted median method allowed for the presence of invalid instrumental variables and reduced type I error occurrence (Hartwig et al., 2017). In our analysis, we primarily used the IVW method as the main analysis method, while the other four statistical methods were used as secondary references. When the *p*-value of the IVW method was less than 0.05 and its direction (positive or negative) was consistent with the other four statistics, we considered that there was a causal relationship (Wang et al., 2023).

Furthermore, sensitivity analysis is essential for detecting heterogeneity and horizontal pleiotropy in MR analysis as well as for assessing the robustness of the results. Cochran’s *Q* test was applied to determine whether SNPs were heterogeneous and MR-PRESSO and MR-Egger regression tests was applied to detect the potential horizontal pleiotropy. Then we performed “leave-one-out” analysis which excluded one SNP at a time to test the stability of our results (Verbanck et al., 2018). Additionally, we verified that the selected SNPs were not associated with common risk factors for ED (diabetes, smoking, and endocrine disorders) using the Phenoscanner website.^1^

All of the above analyses were mainly performed using the TwoSampleMR package (version 0.5.5) in R software (version 4.0.1). MR estimates with *p* < 0.05 were considered nominally significant in this study. For other reported tests in this study, *p* < 0.05 was considered statistically significant.

## 3 Results

### 3.1 Instrumental variable selection

Based on the selection criteria (*p* < 1 × 10^−5^, *R*^2^ < 0.001, clumping distance = 10,000 kb), we identified 2,564 SNPs as IVs for 211 taxonomic groups of gut microbiota. The *F* values for all SNPs were greater than 10, indicating there was no weak instrumental bias in our IVs. Detailed information on the IVs can be found in Supplementary Table S2.

### 3.2 Causal relationship between gut microbiota and ED

In MR analysis, we performed IVW method, MR Egger, simple mode, weighted median, and weighted mode for causal analysis. Detailed analysis results are presented in Supplementary Table S3.

**Figure 2:**
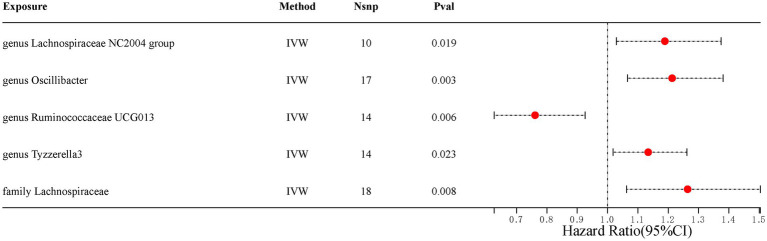
Scatterplot of causal association between gut microbiota and ED. (Each black dot represents an SNP, and the slopes of the straight lines represent potential causality for each method. A: MR Test of Genus Lachnospiraceae NC2004 group; B: MR Test of Genus Oscillibacter C: MR Test of Genus Ruminococcaceae UCG013 D: MR Test of Genus Tyzzerella3 E: MR Test of Family Lachnospiraceae.

Our analysis identified five gut microbiota taxa that were causally associated with ED (Table 1). The risk forest plot showed a negative causal relationship between genus Ruminococcaceae UCG013 and ED (OR = 0.761, 95% CI 0.626–0.926), while family Lachnospiraceae, genus Lachnospiraceae NC2004 group, genus Oscillibacter, and genus Tyzzerella3 were associated with an increased risk of ED, with the family Lachnospiraceae having the highest risk (OR = 1.264, 95% CI 1.063–1.504) (Figure 2).

**Table 1:** Significant results of MR analysis between gut microbiota and ED.

Exposure	Method	*p*-value	OR	95% CI
Genus Lachnospiraceae NC2004 group	IVW	0.019	1.189	1.029–1.374
	MR Egger	0.282	1.438	0.775–2.667
	Simple mode	0.102	1.369	0.976–1.921
	Weighted median	0.011	1.279	1.059–1.545
	Weighted mode	0.087	1.362	0.994–1.868
Genus Oscillibacter	IVW	0.003	1.213	1.066–1.381
	MR Egger	0.447	1.209	0.751–1.945
	Simple mode	0.163	1.294	0.916–1.826
	Weighted median	0.067	1.186	0.988–1.424
	Weighted mode	0.232	1.230	0.887–1.704
Genus Ruminococcaceae UCG013	IVW	0.006	0.761	0.626–0.926
	MR Egger	0.073	0.576	0.332–0.998
	Simple mode	0.110	0.715	0.486–1.050
	Weighted median	0.014	0.721	0.555–0.936
	Weighted mode	0.096	0.718	0.499–1.031
Genus Tyzzerella3	IVW	0.023	1.134	1.018–1.262
	MR Egger	0.811	1.079	0.586–1.988
	Simple mode	0.160	1.220	0.939–1.584
	Weighted median	0.054	1.159	0.997–1.347
	Weighted mode	0.162	1.220	0.938–1.586
Family Lachnospiraceae	IVW	0.008	1.264	1.063–1.504
	MR Egger	0.089	1.496	0.967–2.314
	Simple mode	0.188	1.357	0.877–2.098
	Weighted median	0.026	1.318	1.033–1.680
	Weighted mode	0.085	1.363	0.978–1.900

**Figure 3:**
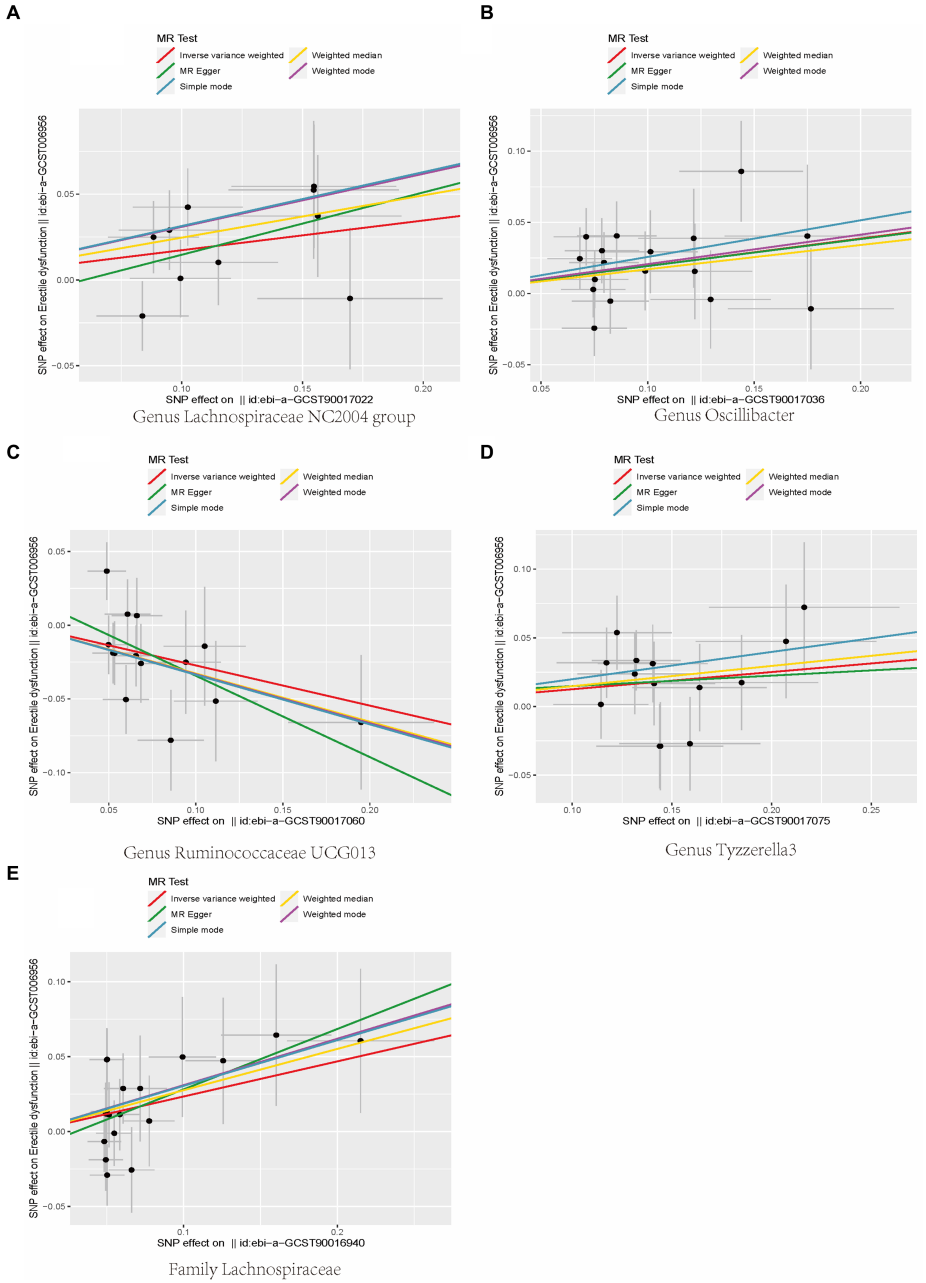
Scatterplot of causal association between gut microbiota and ED. (Each black dot represents an SNP, and the slopes of the straight lines represent potential causality for each method. **(A)** MR Test of Genus Lachnospiraceae NC2004 group; **(B)** MR Test of Genus Oscillibacter **(C)** MR Test of Genus Ruminococcaceae UCG013 **(D)** MR Test of Genus Tyzzerella3 **(E)** MR Test of Family Lachnospiraceae.

In addition, the scatter plot showed a consistent direction of all five statistical methods, confirming the reliability of the results in our study (Figure 3).

### 3.3 Sensitivity analysis

**Figure 4:**
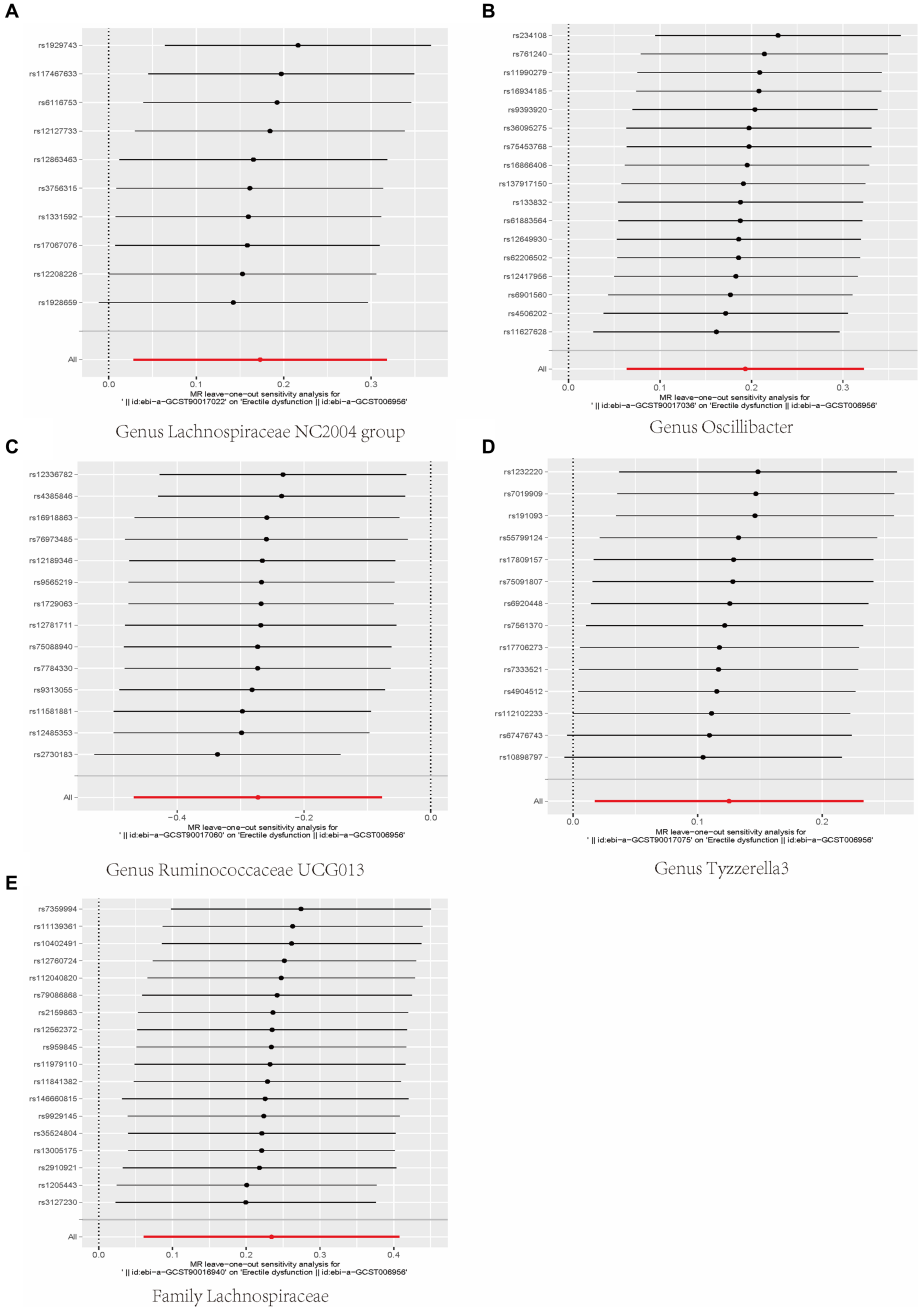
Leave-one-out analysis of the causal relationship between gut microbiota and ED. Scatterplot of causal association between gut microbiota and ED. (Each black dot represents an SNP, and the slopes of the straight lines represent potential causality for each method. **(A)** MR Test of Genus Lachnospiraceae NC2004 group; **(B)** MR Test of Genus Oscillibacter **(C)** MR Test of Genus Ruminococcaceae UCG013 **(D)** MR Test of Genus Tyzzerella3 **(E)** MR Test of Family Lachnospiraceae.

Cochran’s *Q* test indicated that all positive results have *Q* values greater than 0.05. Both MR-Egger intercept and MR-PRESSO test showed that *p*-values for all positive results are greater than 0.05, which indicated that there was no heterogeneity and horizontal pleiotropy in our results. Detailed data can be found in Supplementary Table S4. Finally, we further confirmed that all positive results were reliable using leave-one-out analysis (Figure 4).

## 4 Discussion

ED is very common in adult men and increases with age. As society ages, the prevalence of ED is on the rise. It is estimated that there are nearly 150 million ED patients worldwide, and this number may reach 350 million in the near future (Burnett et al., 2018). The cause of ED is unclear and may be due to organic (vascular, neurogenic, hormonal, etc.) factors, psychosomatic or mixed causes (Bovijn et al., 2019). In this study, we investigated the causal relationship between gut microbiota and male ED using MR analysis. Our findings revealed a positive causal association between the genus Lachnospiraceae NC2004 group, genus Oscillibacte, genus Tyzzerella3, and the family Lachnospiraceae, and male ED. On the other hand, we observed a negative causal relationship between the genus Ruminococcaceae UCG013 and ED. These results suggest that different gut microbial communities may have different effects on male ED. This research provides the first conclusive evidence of a causal relationship between gut microbiota and ED, and provides a new direction for future interventions targeting the gut microbiota in the treatment of ED.

The gut microbiota includes commensal, opportunistic and pathogenic bacteria in the human gastrointestinal tract. The gut microbiota contains more than 100 times the amount of genes as the human genome, and has therefore been referred to as the “second genome” and “eighth organ” of the human body (Baquero and Nombela, 2012). The gut microbiota plays a crucial role in human health and diseases. It has been found that gut microbiota and its metabolites, such as short-chain fatty acids and amino acids, can affect glucose and lipid metabolism through a variety of pathways, which in turn can affect the function of penile vascular endothelial cells (Wu et al., 2021; Mitidieri et al., 2020). Some studies have also found that gut microbiota imbalance will affect the content of NO, Lactobacillus and Bifidobacterium were the most efficient NO-producing strains, whereas *Escherichia coli* and Bacteroides polymorphicus may deplete NO (Sobko et al., 2006; Vermeiren et al., 2009). NO, in turn, had an important impact on the brain’s libido, the blood supply to the testes, and the release of sex hormones. This suggested that gut microbiota can affect erectile function in men through a variety of ways. Our results suggest that modulation of the gut microbiota may be a potential approach to treat or improve ED, and that gut microbial markers may also have the potential to predict the development of ED and serve as effective indicators for ED prevention. In the future, the gut flora of ED patients can be effectively improved in various ways, such as gut flora transplantation, to restore the patient’s intestinal microcosm, which in turn can have a therapeutic effect.

Lachnospiraceae is one of the core gut microbiota, which exists in the gut of most healthy individuals and is involved in the metabolism of various carbohydrates, producing acetate and butyrate through fermentation to provide energy to the host (Stackebrandt, 2014). However, controversy still exists regarding whether Lacinospora is a beneficial or harmful microorganism, possibly due to the different roles played by its various components at different stages and environments (Vacca et al., 2020). In the Canadian Healthy Infant Longitudinal Development (CHILD) study, a decrease in the relative abundance of Lachnospiraceae at 3 months of age was found to be associated with asthma. Moreover, the ratio of Lachnospira/Clostridium was identified as a potential biomarker for predicting asthma development (Stiemsma et al., 2016). Another study discovered that *Lachnospira multipara* and *Eubacterium eligens* consistently decreased throughout the progression from early to advanced stages of colon cancer (Zhang et al., 2023). Research by Shen et al. (2017) revealed a significant increase in the abundance of Lachnospiraceae in populations with non-alcoholic liver cirrhosis and severe liver fibrosis. Other studies also found a positive relationship (*p* < 0.05) between Lachnospiraceae and severe major depressive disorder (MDD) (Vacca et al., 2020). Similarly, our study identified a positive causal relationship (OR = 1.264, *p* < 0.05) between Lachnospiraceae and ED. Subgroups within Lachnospiraceae, namely genus Lachnospiraceae NC2004 group and genus Tyzzerella3 also showed positive causal relationships with ED, with OR of 1.189 and 1.213, respectively. These findings suggest that Lachnospiraceae may play a crucial role in male ED, but the specific mechanisms need to be further investigated.

Ruminococcaceae is also one of the cornerstone bacteria in the gut, playing a significant role in metabolism and normal physiological activities. A meta-analysis revealed a significant decrease in the abundance of Ruminococcaceae in populations with cognitive impairments and Alzheimer’s disease (Chen et al., 2023). Another meta-analysis similarly found a significant decrease in the abundance of Ruminococcus ruminococcus in a bipolar disorder population (Nguyen et al., 2021). Feng et al. (2022) found that Ruminococcaceae_UCG-013 was positively correlated with serum HDL cholesterol levels and negatively correlated with serum total cholesterol, total cholesterol, and LDL cholesterol levels. In our study, we found a negative causal relationship between Ruminococcaceae_UCG-013 and ED (OR = 0.761, 95% CI 0.626–0.926). This suggests that Ruminococcaceae_UCG-013 may affect erectile through lipid metabolism pathway.

However, our study also has several limitations. Firstly, all the GWAS data involved in our research were obtained from European populations. Therefore, further validation is needed to assess the applicability of our research findings to other racial and ethnic groups. Secondly, the sample size in our study was relatively small, which may introduce biases and chance effects, and therefore needs to be validated by larger population-based GWAS studies. Finally, when selecting SNPs for exposure factor, we used a more relaxed threshold (*p* < 1 × 10^−5^) than the traditional threshold (*p* < 5 × 10^–8^). This may have led to the inclusion of SNPs with weaker correlations. However, we found that each instrumental variable was greater than 10 through *F*-test.

## 5 Conclusion

We comprehensively assessed the causal relationship between gut microbiota and ED in men. Our findings indicated that four gut microbiota were positively causally associated with ED and one gut microbiota was negatively causally associated with ED. Our study provides new insights into the involvement of gut microbiota in the occurrence and development of ED.

## Data Availability

The datasets presented in this study can be found in online repositories. The names of the repository/repositories and accession number(s) can be found in the article/Supplementary material.

## Footnotes

^1^ http://www.phenoscanner.medchsl.cam.ac.uk/
