# Supplementary material for: Causal relationship between gut microbiota and male erectile dysfunction: a Mendelian randomization analysis
Source: Front Microbiol. 2024 Aug 29;15:1367740. doi: 10.3389/fmicb.2024.1367740 (PMC11390668; doi:10.3389/fmicb.2024.1367740)
Supplement: Supplementary file 1 [file Table_1.docx]

**STROBE-MR checklist of recommended items to address in reports of Mendelian randomization studies**

| **Item No.** | **Section** | **Checklist item** | **Relevant text from manuscript** |
| --- | --- | --- | --- |
| 1 | **TITLE and ABSTRACT** | Indicate Mendelian randomization (MR) as the study’s design in the title and/or the abstract if that is a main purpose of the study | Causal Relationship Between Gut Microbiota and Male Erectile Dysfunction: A Mendelian Randomization Analysis |
|  | **INTRODUCTION** |  |  |
| 2 | **Background** | Explain the scientific background and rationale for the reported study. What is the exposure? Is a potential causal relationship between exposure and outcome plausible? Justify why MR is a helpful method to address the study question | Several observational studies have reported an association between gut microbiota and male erectile dysfunction (ED). However, it remains unclear whether there is a causal relationship between gut microbiota and male ED. Thus, we aimed to investigate the potential causal relationship between gut microbiota and male ED using Mendelian randomization (MR) analysis. |
| 3 | **Objectives** | State specific objectives clearly, including pre-specified causal hypotheses (if any). State that MR is a method that, under specific assumptions, intends to estimate causal effects | To assess the causal relationship between gut microbiota and male ED, we conducted a two-sample Mendelian randomization (MR) analysis. |
|  | **METHODS** |  |  |
| 4 | **Study design and data sources** | Present key elements of the study design early in the article. Consider including a table listing sources of data for all phases of the study. For each data source contributing to the analysis, describe the following: |  |
|  | a) | Setting: Describe the study design and the underlying population, if possible. Describe the setting, locations, and relevant dates, including periods of recruitment, exposure, follow-up, and data collection, when available. | 2.1 Data Sources and Figure1 |
|  | b) | Participants: Give the eligibility criteria, and the sources and methods of selection of participants. Report the sample size, and whether any power or sample size calculations were carried out prior to the main analysis | The statistical data of gut microbiota were obtained from 16S rRNA gene sequencing profiles and genotype data provided by MiBioGen, including a total of 18,340 participants from 24 cohorts, most of them were of European descent (13,266 individuals). The date encompassed 211 taxa, including 131 genera, 35 families, 20 orders, 16 classes, and 9 phyla. The GWAS data for erectile dysfunction were obtained from the study conducted by Jonas et al. in 2019, which included 6,175 cases and 217,630 controls from the Partners HealthCare Biobank, the Estonian Genome Center of the University of Tartu, and the UK Biobank, all of them were of European descent. |
|  | c) | Describe measurement, quality control and selection of genetic variants | 1) a relatively lenient significance threshold of p < 1×10^-5 was used due to the limited number of SNPs with genome-wide significance. 2) SNPs that violate linkage disequilibrium (LD) criteria (R^2 < 0.001, cluster distance = 10,000 kb) were excluded. 3) The F-statistic was calculated to assess the strength of SNPS, If the corresponding F-statistic was >10, it was considered that there was no significant weak instrumental bias. The formula for calculating F as follows: F = R2 × (n−1−k) / (1−R2) × k. R2 represented the proportion of variance in the exposure explained by the genetic variants, N represented sample size, and K represented the number of instruments. [20] 4) SNPs with inconsistent alleles between exposure and outcome (e.g. A/G and A/C) and palindromic A/T or G/C alleles were excluded. |
|  | d) | For each exposure, outcome, and other relevant variables, describe methods of assessment and diagnostic criteria for diseases | ED was defined as self-reported or physician-reported ED (ICD10 codes N48.4 and F52.2), or use of ED medications (sildenafil/Viagra, tadalafil/Cialis, or vardenafil/Levitra), or history of ED surgery (OPCS-4 codes L97.1 and N32.6). |
|  | e) | Provide details of ethics committee approval and participant informed consent, if relevant | We performed a two-sample MR analysis in strict accord with the requirements of the STORBE-MR guidelines, details were provided in Table S1. |
| 5 | **Assumptions** | Explicitly state the three core IV assumptions for the main analysis (relevance, independence and exclusion restriction) as well assumptions for any additional or sensitivity analysis | The GWAS data were all approved by the Ethics Committee in the original language and therefore did not need to be obtained additionally. |
| 6 | **Statistical methods: main analysis** | Describe statistical methods and statistics used |  |
|  | a) | Describe how quantitative variables were handled in the analyses (i.e., scale, units, model) | We employed five Mendelian randomization (MR) analysis methods, including Inverse Variance Weighted (IVW) method, MR-Egger method, Weighted Median method, Weighted Mode method, and Simple Mode method, to assess the causal relationship between gut microbiota and erectile dysfunction. |
|  | b) | Describe how genetic variants were handled in the analyses and, if applicable, how their weights were selected | In our analysis, we primarily used the IVW method as the main analytical approach, while the other four statistical methods were used as secondary references. |
|  | c) | Describe the MR estimator (e.g. two-stage least squares, Wald ratio) and related statistics. Detail the included covariates and, in case of two-sample MR, whether the same covariate set was used for adjustment in the two samples | IVW combined the Wald ratio estimates of each instrumental variable in a weighted linear regression of instrumental variables on the outcome. |
|  | d) | Explain how missing data were addressed | YES |
|  | e) | If applicable, indicate how multiple testing was addressed |  |
| 7 | **Assessment of assumptions** | Describe any methods or prior knowledge used to assess the assumptions or justify their validity | MR-Egger method was based on the InSIDE (instrument strength independent of direct effect) assumption, accounting for the presence of pleiotropy. The Weighted Median method allowed for the presence of invalid instrumental variables and reduced Type I error occurrence. |
| 8 | **Sensitivity analyses and additional analyses** | Describe any sensitivity analyses or additional analyses performed (e.g. comparison of effect estimates from different approaches, independent replication, bias analytic techniques, validation of instruments, simulations) | We employed Cochran's Q statistic, MR-Egger intercept test, MR-PRESSO, and leave-one-out analysis to detect heterogeneity, and horizontal pleiotropy, and evaluate the robustness of the results |
| 9 | **Software and pre-registration** |  |  |
|  | a) | Name statistical software and package(s), including version and settings used | TwoSampleMR package (version 0.5.5) in R software (version 4.0.1). |
|  | b) | State whether the study protocol and details were pre-registered (as well as when and where) |  |
|  | **RESULTS** |  |  |
| 10 | **Descriptive data** |  |  |
|  | a) | Report the numbers of individuals at each stage of included studies and reasons for exclusion. Consider use of a flow diagram | Figure1 |
|  | b) | Report summary statistics for phenotypic exposure(s), outcome(s), and other relevant variables (e.g. means, SDs, proportions) | where gut microbiota is considered as the exposure factor and erectile dysfunction as the outcome. The statistical data of gut microbiota were obtained from 16S rRNA gene sequencing profiles and genotype data provided by MiBioGen, including a total of 18,340 participants from 24 cohorts, most of them were of European descent (13,266 individuals). The date encompassed 211 taxa, including 131 genera, 35 families, 20 orders, 16 classes, and 9 phyla . The GWAS data for erectile dysfunction were obtained from the study conducted by Jonas et al. in 2019, which included 6,175 cases and 217,630 controls from the Partners HealthCare Biobank, the Estonian Genome Center of the University of Tartu, and the UK Biobank, all of them were of European descent. |
|  | c) | If the data sources include meta-analyses of previous studies, provide the assessments of heterogeneity across these studies | We employed Cochran's Q statistic, MR-Egger intercept test, MR-PRESSO, and leave-one-out analysis to detect heterogeneity |
|  | d) | For two-sample MR:  i.  Provide justification of the similarity of the genetic variant-exposure associations between the exposure and outcome samples  ii.  Provide information on the number of individuals who overlap between the exposure and outcome studies | Additionally, we verified that the selected SNPs were not associated with common risk factors for erectile dysfunction (diabetes, smoking, and endocrine disorders) using the Phenoscanner website(http://www.phenoscanner.medchsl.cam.ac.uk/). |
| 11 | **Main results** |  |  |
|  | a) | Report the associations between genetic variant and exposure, and between genetic variant and outcome, preferably on an interpretable scale | Based on the selection criteria (p < 0.00001, R2 < 0.01, clumpingr distance = 10,000 kb), we identified 2564 SNPs as instrumental variables (IVs) for 211 taxonomic groups of gut microbiota. The F values for all SNPs were greater than 10, indicating the absence of weak instrument bias in our instrumental variable set |
|  | b) | Report MR estimates of the relationship between exposure and outcome, and the measures of uncertainty from the MR analysis, on an interpretable scale, such as odds ratio or relative risk per SD difference | We considered a causal relationship to exist when the p-value of the IVW (inverse variance-weighted) method was <0.05, and its direction (positive or negative) aligned with the other four statistical results. Detailed analysis results are presented in Table S3. |
|  | c) | If relevant, consider translating estimates of relative risk into absolute risk for a meaningful time period | Table1 Positive results of the gut microbiota and male erectile dysfunction (ED) Mendelian Randomization (MR) analysis |
|  | d) | Consider plots to visualize results (e.g. forest plot, scatterplot of associations between genetic variants and outcome versus between genetic variants and exposure) | Figure2 Effect estimates for association of genetically predicted gut microbiota with erectile dysfunction risk using inverse variance weighted method |
| 12 | **Assessment of assumptions** |  |  |
|  | a) | Report the assessment of the validity of the assumptions |  |
|  | b) | Report any additional statistics (e.g., assessments of heterogeneity across genetic variants, such as *I^2^*, Q statistic or E-value) | Cochran's Q test indicates that all positive results have Q values greater than 0.05 |
| 13 | **Sensitivity analyses and additional analyses** |  |  |
|  | a) | Report any sensitivity analyses to assess the robustness of the main results to violations of the assumptions | Both MR-Egger and MR-PRESSO analyses show that MR egger_interpreter and MRPRESS_GLOBAL p-values for all positive results are greater than 0.05, indicating the absence of heterogeneity and horizontal pleiotropy in our results |
|  | b) | Report results from other sensitivity analyses or additional analyses | MR-Egger, MR-PRESSO, MR egger_interpreter, MRPRESS_GLOBAL |
|  | c) | Report any assessment of direction of causal relationship (e.g., bidirectional MR) |  |
|  | d) | When relevant, report and compare with estimates from non-MR analyses |  |
|  | e) | Consider additional plots to visualize results (e.g., leave-one-out analyses) | Figure4 MR leave-one-out analysis. |
|  | **DISCUSSION** |  |  |
| 14 | **Key results** | Summarize key results with reference to study objectives | We comprehensively assessed the causal relationships between gut microbiota and male erectile dysfunction. Our findings indicate that four gut microbiotas show a positive causal relationship with male erectile dysfunction, while one gut microbiota exhibits a negative causal relationship. |
| 15 | **Limitations** | Discuss limitations of the study, taking into account the validity of the IV assumptions, other sources of potential bias, and imprecision. Discuss both direction and magnitude of any potential bias and any efforts to address them | However, our study also has several limitations. Firstly, all the GWAS data involved in our research were obtained from European populations. Lastly, in the selection of instrumental variables (SNPs) for exposure factors, we employed a more lenient threshold (p < 1×10−5) instead of the conventional threshold (p < 5×10−8). |
| 16 | **Interpretation** |  |  |
|  | a) | Meaning: Give a cautious overall interpretation of results in the context of their limitations and in comparison with other studies | Therefore, further validation is needed to assess the applicability of our research findings to other racial and ethnic groups. Secondly, the sample size in our study was relatively small, which may introduce biases and chance effects, necessitating validation through larger population-based GWAS studies |
|  | b) | Mechanism: Discuss underlying biological mechanisms that could drive a potential causal relationship between the investigated exposure and the outcome, and whether the gene-environment equivalence assumption is reasonable. Use causal language carefully, clarifying that IV estimates may provide causal effects only under certain assumptions |  |
|  | c) | Clinical relevance: Discuss whether the results have clinical or public policy relevance, and to what extent they inform effect sizes of possible interventions | This research provides the first conclusive evidence of a causal link between gut microbiota and erectile dysfunction, offering new directions for future interventions targeting gut microbiota to treat erectile dysfunction. |
| 17 | **Generalizability** | Discuss the generalizability of the study results (a) to other populations, (b) across other exposure periods/timings, and (c) across other levels of exposure | Current research on the gut microbiota mainly focuses on areas such as the nervous system, tumors, and metabolism. Studies investigating the impact of the gut microbiota on the reproductive system are relatively scarce and predominantly observational. For instance, one study compared the fecal and semen samples of mice fed a high-fat diet with those of a control group, revealing that the high-fat diet group experienced a decline in semen quality, accompanied by elevated levels of Rikenellaceae in the feces. |
|  | **OTHER INFORMATION** |  |  |
| 18 | **Funding** | Describe sources of funding and the role of funders in the present study and, if applicable, sources of funding for the databases and original study or studies on which the present study is based | The authors received no funding for this work. |
| 19 | **Data and data sharing** | Provide the data used to perform all analyses or report where and how the data can be accessed, and reference these sources in the article. Provide the statistical code needed to reproduce the results in the article, or report whether the code is publicly accessible and if so, where | The dataset provided in this study can be found in an online repository. |
| 20 | **Conflicts of Interest** | All authors should declare all potential conflicts of interest | The authors declare that the research was conducted in the absence of any commercial or financial relationships that could be construed as a potential conflict of interest. |

This checklist is copyrighted by the Equator Network under the Creative Commons Attribution 3.0 Unported (CC BY 3.0) license.

1. Skrivankova VW, Richmond RC, Woolf BAR, Yarmolinsky J, Davies NM, Swanson SA, et al. Strengthening the Reporting of Observational Studies in Epidemiology using Mendelian Randomization (STROBE-MR) Statement. JAMA. 2021;under review.

2. Skrivankova VW, Richmond RC, Woolf BAR, Davies NM, Swanson SA, VanderWeele TJ, et al. Strengthening the Reporting of Observational Studies in Epidemiology using Mendelian Randomisation (STROBE-MR): Explanation and Elaboration. BMJ. 2021;375:n2233.

1. Skrivankova VW, Richmond RC, Woolf BAR, Yarmolinsky J, Davies NM, Swanson SA, et al. Strengthening the Reporting of Observational Studies in Epidemiology using Mendelian Randomization (STROBE-MR) Statement. JAMA. 2021;under review.

2. Skrivankova VW, Richmond RC, Woolf BAR, Davies NM, Swanson SA, VanderWeele TJ, et al. Strengthening the Reporting of Observational Studies in Epidemiology using Mendelian Randomisation (STROBE-MR): Explanation and Elaboration. BMJ. 2021;375:n2233.
